# The protective effect of antioxidants on orbital fibroblasts from patients with Graves’ ophthalmopathy in response to oxidative stress

**Published:** 2013-04-16

**Authors:** Chieh-Chih Tsai, Shi-Bei Wu, Shu-Ching Kao, Hui-Chuan Kau, Fenq-Lih Lee, Yau-Huei Wei

**Affiliations:** 1 Department of Ophthalmology, Taipei Veterans General Hospital and National Yang-Ming University, Taipei, Taiwan; 2 Department of Biochemistry and Molecular Biology, National Yang-Ming University, Taipei, Taiwan; 3 Department of Ophthalmology, Koo Foundation Sun Yat-Sen Cancer Center, Taipei, Taiwan; 4 Department of Medicine, Mackay Medical College, New Taipei City, Taiwan

## Abstract

Purpose: To investigate the biphasic effects of hydrogen peroxide (H_2_O_2_) on the orbital fibroblasts of patients with Graves’ ophthalmopathy (GO) and the relation to antioxidants and proinflammatory cytokines.

**Methods:**

Proliferation of cultured orbital fibroblasts from patients with GO and normal controls was evaluated in response to various concentrations of H_2_O_2_. The effect of low concentrations of H_2_O_2_ (6.25 μM) on the cellular proliferation and induction of intracellular proinflammatory cytokines, and reactive oxygen species of orbital fibroblasts were assessed. Protective effects of N-acetylcysteine and vitamin C on GO fibroblasts in response to 6.25 μM H_2_O_2_ stimulation were also investigated.

**Results:**

When the GO fibroblasts were exposed to H_2_O_2_ at a concentration of 50 μM or above, significant cytotoxicity was observed. In contrast, lower concentrations of H_2_O_2_ (3.125–25 μM) increased the survival of GO fibroblasts with the peak cellular proliferation at 6.25 μM H_2_O_2_. However, this biphasic effect of H_2_O_2_ on the viability of orbital fibroblasts was not found in normal controls. In addition, 6.25 μM H_2_O_2_ led to significant elevation of the levels of transforming growth factor, beta 1, interleukin-1β, and superoxide anion in GO fibroblasts, but no significant change in the normal controls. Pretreatment with N-acetylcysteine or vitamin C reversed the enhanced proliferation capacity and the induction of transforming growth factor, beta 1, interleukin-1β and superoxide anion of GO fibroblasts in response to 6.25 μM H_2_O_2_.

**Conclusions:**

These findings revealed the biphasic effect of H_2_O_2_ on cellular proliferation of GO orbital fibroblasts. Importantly, a low level of H_2_O_2_ can stimulate proliferation of GO orbital fibroblasts and induce the production of proinflammatory cytokines, which can be inhibited by pretreatment with antioxidants. This provides a theoretical basis for the rational use of antioxidant in treating GO at an early stage.

## Introduction

Graves’ ophthalmopathy (GO), the most important and frequent extrathyroidal expression of Graves’ disease, is an inflammatory disorder of autoimmune background [1,2]. The pathogenesis of GO is thought to be a complex interplay between endogenous and environmental factors [3,4]. Recently, increasing evidence has shown that reactive oxygen species (ROS) play an important role in the development of GO [5]. Elevated extracellular levels of ROS have also been noted in the blood [6], urine [7,8], fibroadipose tissues [9], and orbital fibroblasts [10] of patients with GO. However, the contribution of ROS to the pathogenesis of GO has remained elusive. Hydrogen peroxide (H_2_O_2_), an ROS naturally produced in human cells during physiologic and pathological processes, has been used as a prooxidant in the study of oxidative stress–related diseases. We recently reported that exposure to a sublethal concentration of hydrogen peroxide (200 μM) resulted in marked cytotoxicity and ROS-elicited oxidative damage in GO fibroblasts [11]. However, superoxide anions, one of the main ROS, have been shown to induce proliferation of orbital fibroblasts obtained from two patients with severe Graves' ophthalmopathy in a dose–response manner [12]. In the present study, we investigated the possible biphasic effects of ROS on GO orbital fibroblasts, especially the low-dose effect of ROS and its relation to antioxidants and prooxidant cytokines.

## Methods

### Culture of orbital fibroblasts

The culture of orbital fibroblasts was established from surgical specimens of seven patients with GO during decompression surgery (two men and five women; mean age: 37.6 years) and from apparently normal orbital tissues in five age-matched patients who received surgery for noninflammatory conditions (one man and four women; mean age: 35.2 years). All patients with GO achieved stable euthyroidism for at least 6 months before surgery and were in the inactive stage of GO. In addition, no patients with GO were smokers or ex-smokers and had not received corticosteroid treatment for at least 1 month before surgery. The study was performed according to the tenets of the Declaration of Helsinki, and these activities were approved by the Institutional Review Board of Taipei Veterans General Hospital. Following the protocol used in our previous studies [10,11,13], the orbital tissues were minced aseptically in phosphate-buffered saline (PBS containing 137 mM NaCl, 2.7 mM KCl, 8 mM Na_2_HPO_4_, 1.5 mM KH_2_PO_4_), and then incubated with a sterile solution containing 0.5% collagenase and dispase (Sigma-Aldrich Chemical Co., St. Louis, MO) for 24 h at 37 °C in a culture incubator with an atmosphere of 5% CO_2_. The digested orbital tissues were pelleted by centrifugation at 1,000 g and then resuspended in Dulbecco’s modified eagle’s medium (DMEM; Gibco Life Technologies, Gaithersburg, MD) containing 10% fetal bovine serum (FBS) and antibiotics (Biologic Industries, Kibbutz Beit Haemek, Israel), which was composed of 100 U/ml penicillin G and 100 μg/ml streptomycin sulfate (Biologic Industries). Cultured orbital fibroblasts were used between the third and fifth passages, and the cultures at the same passage number were used for the same set of experiments.

### Analysis of cell proliferation and treatment

About 10^5^ orbital fibroblasts were seeded in 3.5-cm culture dish and incubated for 48 h at 37 °C in a culture incubator with an atmosphere of 5% CO_2_. Cells were then treated with various H_2_O_2_ concentrations (3.125, 6.25, 12.5, 25, 50, and 100 μM) for 24 h or the cells pretreated with N-acetylcysteine (NAC, 100 and 200 μM) or vitamin C (250 and 500 μM), respectively, for 1 h followed by treatment of cells with 6.25 μM H_2_O_2_ for 24 h. To determine cell proliferation, we used the AlamarBlue reagent (AbD Serotec, Oxford, England), which incorporates a fluorometric growth indicator based on the intracellular metabolic activity [14]. Briefly, cells were washed twice with PBS (pH 7.2), and then 1/10 volume of the AlamarBlue reagent was directly added to cells in the culture medium and incubated at 37 °C in a cell incubator with an atmosphere of 5% CO_2_ for 2 h. An aliquot of 200 μl culture medium was then drawn into a 96-well plate, and the fluorescence intensity was measured with the Victor1420 Multilabel Counter (PerkinElmer Life Sciences, Waltham, MA) [2], with the excitation wavelength at 538 nm and emission wavelength at 590 nm.

### Measurement of the intracellular cytokine content

The human transforming growth factor, beta 1 (TGF-β1; catalog #DB100B), interleukin-1β (IL-1β; catalog #DLB50), and tumor necrosis factor alpha (TNF-α; catalog #DTA00C) levels in cell culture supernatant were quantified with enzyme-linked immunosorbent assay kits purchased from R&D Systems, Inc. (Minneapolis, MN). Briefly, about 10^5^ orbital fibroblasts were seeded in a 3.5-cm culture dish and incubated for 48 h at 37 °C in a cell incubator with an atmosphere of 5% CO_2_ followed by treatment of 6.25 μM H_2_O_2_ for another 24 h or the cells were pretreated with NAC (200 μM) or vitamin C (500 μM) for 1 h followed by the treatment of 6.25 μM H_2_O_2_ for 24 h. According to the manufacturer’s recommendation, cell culture supernatant was centrifuged at 12,000 g at 4 °C, and the aliquots were immediately assayed. The standards for TGF-β1, IL-1β, and TNF-α were used in a range of 0–200 pg/ml, and the results were normalized by the cell number and expressed as pg/10^4^ cells.

### Measurement of reactive oxygen species content

According to our previous study, the probes from 2’,7’-dichlorofluorescein diacetate (DCFH-DA, Molecular Probes, Eugene, OR) and dihydroethidine (DHE purchased from Molecular Probes) were used to evaluate the intracellular H_2_O_2_ and O_2_∙^–^ content, respectively [11]. After incubation of orbital fibroblasts with 20 μM DCFH-DA or 10 μM DHE at 37 °C for 20 min, cells were trypsinized and then resuspended in 0.5 ml of PBS buffer (pH 7.4) followed by analysis of flow cytometry with a flow cytometer (Model EPICS XL-MCL, Beckman-Coulter, Miami, FL). The excitation wavelength was set at 488 nm, and the intensity of the emitted fluorescence of a total of 10,000 cells at 525 nm was recorded on channel FL1 for the DCFH-DA probe and at 575 nm was recorded on channel FL2 for the DHE probe, respectively. Data were acquired and analyzed using EXPO32 software (Beckman-Coulter, Miami, FL), and the intracellular H_2_O_2_ or O_2_∙^–^ content in the treated cells is presented as a relative value compared to that of the cells without 6.25 μM H_2_O_2_ or antioxidant treatment (200 μM NAC or 500 μM vitamin C).

### Statistical analysis

Statistical analysis was performed by using the Microsoft Excel 2010 statistical package and SigmaPlot software version 12.3 (Systat Software Inc., San Jose, CA). The data are presented as means ± standard error of the mean (SEM) of the results obtained from three independent experiments. The significance level of the difference between the control and the experimental groups was determined with the Student *t* test. A difference was considered statistically significant when the p value <0.05 and p value <0.01, respectively.

## Results

### Effect of various concentrations of hydrogen peroxide on the viability of orbital fibroblasts

**Figure 1:**
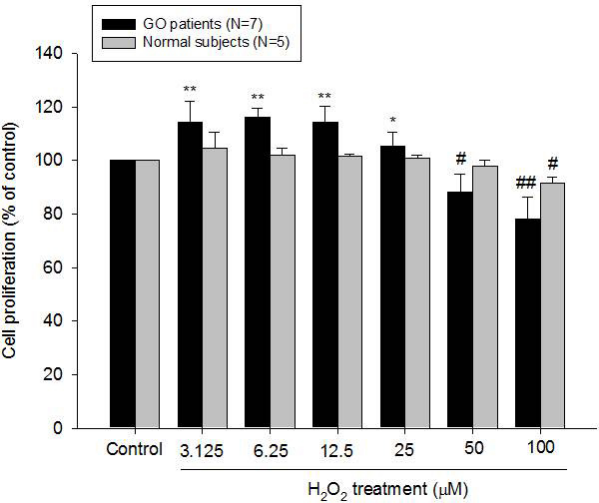
Comparison of the effects of hydrogen peroxide at various concentrations on the viability of orbital fibroblasts between patients with Graves’ ophthalmopathy (GO) and normal controls. We treated orbital fibroblasts from patients with GO (n=7) and age-matched normal subjects (n=5) with various concentrations of hydrogen peroxide (H_2_O_2_) for 24 h. The cell proliferation rate was examined with the AlamarBlue assay as methods described and normalized to each control not exposed to H_2_O_2_. The mean values of cell proliferation in H_2_O_2_-treated orbital fibroblasts are shown in the histogram. Treatment with a low concentration of H_2_O_2_ (<25 μM) in GO orbital fibroblasts significantly induced the cell proliferation, but the effect was not observed in normal subjects. The cytotoxicity of H_2_O_2_ was observed in GO orbital fibroblasts above 50 μM and in normal subjects above 100 μM. The data are presented as mean ± standard deviation of the results from three independent experiments. (Significant increase when ** p<0.01 and *p<0.05; significant decrease when ##p<0.01 and #p<0.05.)

The effect of H_2_O_2_ on the viability of orbital fibroblasts, as determined with the AlamarBlue cell viability assay, is illustrated in Figure 1. The data show that there was a biphasic effect of H_2_O_2_ on the viability of GO orbital fibroblasts. Cytotoxicity was not observed in the concentration range of 3.125–25 μM H_2_O_2_ when the GO fibroblasts were incubated with H_2_O_2_ for 24 h. In contrast, lower concentrations of H_2_O_2_ increased the survival of GO orbital fibroblasts with the peak proliferation (mean increase: 16.4%) at 6.25 μM H_2_O_2_ (p=0.0001). When the GO fibroblasts were exposed to H_2_O_2_ at a concentration of 50 μM or above, significant cytotoxicity was observed (p=0.0038). Different from GO orbital fibroblasts, control orbital fibroblasts showed no significant proliferation in response to low concentrations of H_2_O_2_ (3.125–25 μM). Cell cultures of normal controls exposed to H_2_O_2_ at a concentration above 100 μM started to reveal significant cytotoxicity (p=0.0011).

### Low concentration of hydrogen peroxide–induced changes of intracellular cytokines in orbital fibroblasts

The changes in the intracellular cytokines upon treatment of orbital fibroblasts with 6.25 μM H_2_O_2_ are shown in Table 1. Basal levels of TGF-β1 and IL-1β were significantly higher in the GO orbital fibroblasts compared with those of the control group (p<0.001 and p<0.001, respectively). Low concentrations of H_2_O_2_ led to significant elevation in TGF-β1 and IL-1β levels in GO orbital fibroblasts compared with the respective controls (p<0.001 and p=0.005, respectively). In addition, the induction ratio of TGF-β1 and IL-1β after treatment with a low dose of H_2_O_2_ were more pronounced in the GO orbital fibroblasts than those in the normal controls (p<0.001 and p<0.001, respectively). These findings were not observed in TNF-α in the GO orbital fibroblasts. Conversely, there was no significant increase in the intracellular levels of TNF-α, TGF-β1, and IL-1β in the normal controls after treatment with low levels of H_2_O_2_.

**Table 1:** The expression levels of intracellular cytokines in orbital fibroblasts before and after treatment of the cells with 6.25 μM H_2_O_2_.

Cytokine species	Before treatment	After treatment	Induction ratio (%)*	p -value
	(mean ± SD)	(mean ± SD)	(mean ± SD)	
**TNF-*α (pg per 10^4^ cells)***				
Normal	32.16±5.36	35.02±6.17	108.71±5.07	0.881
GO	31.60±5.15	33.73±9.39	106.44±8.13	0.603
	p=0.755		p=0.705	
**TGF-*β1 (pg per 10^4^ cells)***				
Normal	95.73±10.71	92.08±12.88	96.17±11.73	0.631
GO	126.61±15.04	164.82±18.83	130.15±18.64	<0.001
	p<0.001		p<0.001	
**IL-1*β (pg per 10^4^ cells)***				
Normal	43.32±5.85	45.07±3.14	104.62±8.84	0.537
GO	52.86±4.13	62.59±5.57	123.18±12.90	0.005
	p<0.001		p<0.001	

* Induction ratio=the expression of cytokine value after H_2_O_2_ treatment / baseline value (%)

### Modulation of low concentration hydrogen peroxide–induced cellular proliferation and changes of intracellular levels of transforming growth factor, beta 1 and interleukin-1β in Graves’ ophthalmopathy orbital fibroblasts with various antioxidants

**Figure 2:**
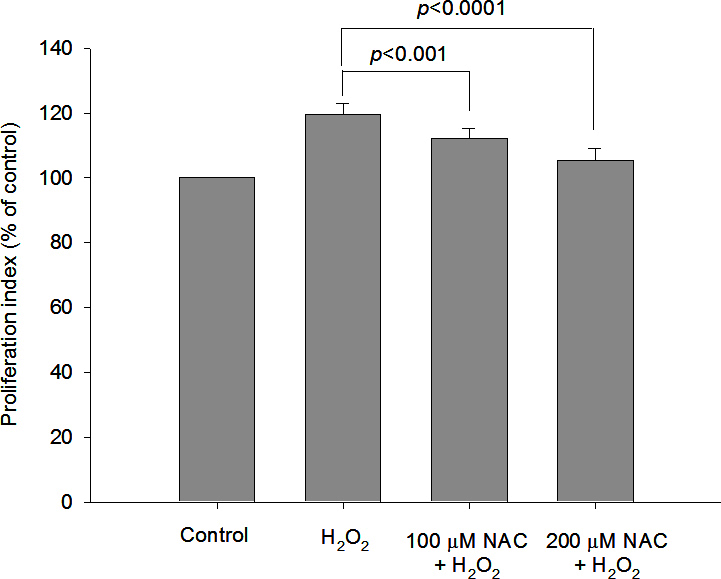
Protective effect of N-acetylcysteine against 6.25 μM hydrogen peroxide–induced proliferation of orbital fibroblasts from patients with Graves’ ophthalmopathy. After pretreatment of Graves’ ophthalmopathy (GO) orbital fibroblasts (n=7) with 100 μM or 200 μM N-acetylcysteine (NAC) for 1 h, followed by the addition of 6.25 μM hydrogen peroxide (H_2_O_2_) for 24 h, the cell proliferation rate was examined with the AlamarBlue assay. The data were normalized to each control that was not exposed to H_2_O_2_, and the mean values of cell proliferation are shown in the histogram. The pretreatment of NAC at 100 μM and 200 μM in GO orbital fibroblasts significantly abolished H_2_O_2_-induced cell proliferation. The data are presented as mean ± standard deviation of the results from three independent experiments. (p<0.001 and p<0.0001 represented significant decrease.)

**Figure 3:**
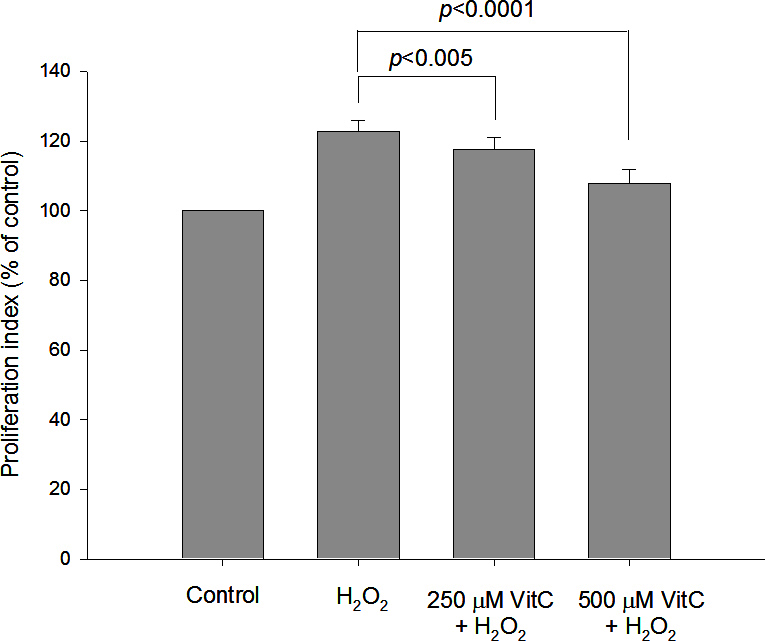
Protective effect of vitamin C against 6.25 μM hydrogen peroxide–induced proliferation of orbital fibroblasts from patients with Graves’ ophthalmopathy. After pretreatment of Graves’ ophthalmopathy (GO) orbital fibroblasts (n=7) with 250 μM or 500 μM vitamin C (VitC) for 1 h, followed by the addition of 6.25 μM hydrogen peroxide (H_2_O_2_) for 24 h, the cell proliferation rate was examined with the AlamarBlue assay. The data were normalized to each control not exposed to H_2_O_2_, and the mean values of cell proliferation from GO orbital fibroblasts are shown in the histogram. The pretreatment of VitC at 250 μM and 500 μM in GO orbital fibroblasts significantly inhibited H_2_O_2_-induced cell proliferation. The data are presented as mean ± standard deviation of the results from three independent experiments. (p<0.005 and p<0.0001 represented significant decrease.)

**Figure 4:**
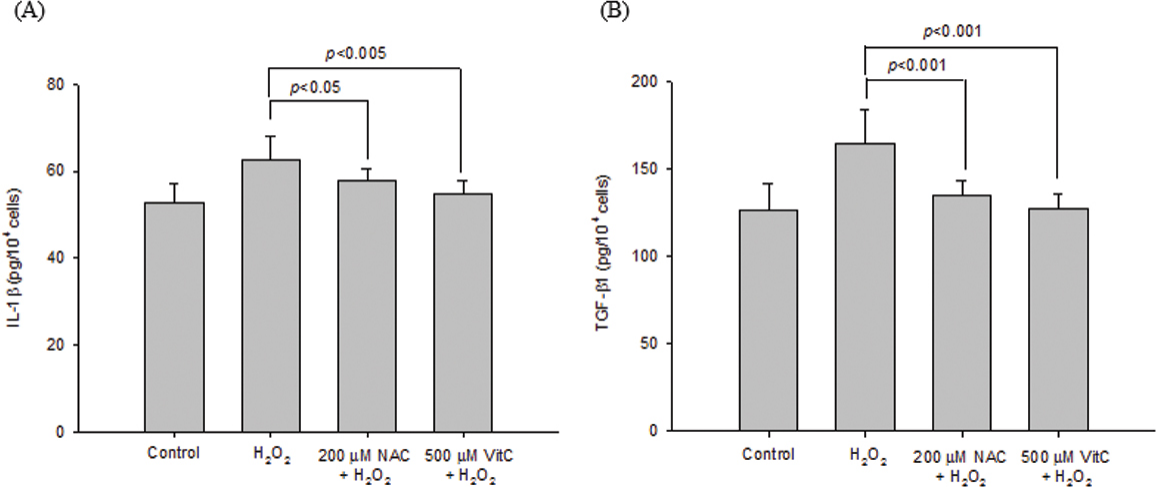
Protective effect of N-acetylcysteine and vitamin C against hydrogen peroxide–induced expression of intracellular levels of interleukin-1β and transforming growth factor, beta 1 in Graves’ ophthalmopathy orbital fibroblasts. After pretreatment of Graves’ ophthalmopathy (GO) orbital fibroblasts (n=7) with 200 μM N-acetylcysteine (NAC) or 500 μM vitamin C (VitC) for 1 h, followed by the addition of 6.25 μM hydrogen peroxide (H_2_O_2_) for 24 h, the release of the intracellular levels of interleukin-1β (IL-1β) and transforming growth factor, beta 1 (TGF-β1) was determined with an enzyme-linked immunosorbent assay kit. No H_2_O_2_ treatment in GO orbital fibroblasts was represented as the control (basal level). The mean values of IL-1β and TGF-β1 from the GO orbital fibroblasts are shown in the histogram (**A** and **B**, respectively). The H_2_O_2_-induced release of the IL-1β and TGF-β1 levels in the GO orbital fibroblasts was significantly abolished by pretreatment with 200 μM NAC or 500 μM VitC, respectively. The data are presented as mean ± standard deviation of the results from three independent experiments. (p<0.05, p<0.005, and p<0.001 represented significant decrease.)

Figure 2 and Figure 3 show the protective effects of NAC and vitamin C, respectively, in GO orbital fibroblast proliferation in response to 6.25 μM H_2_O_2_. Preincubation with 100 μM or 200 μM NAC significantly decreased H_2_O_2_-induced GO orbital fibroblast proliferation (p<0.001 and p<0.0001, respectively). A significant reduction in H_2_O_2_-induced fibroblast proliferation was also obtained after the cells were preincubated with 250 μM or 500 μM vitamin C (p=0.0048 and p<0.0001, respectively). Figure 4 demonstrates the protective effects of NAC and vitamin C against 6.25 μM H_2_O_2_-induced expression of intracellular IL-1β and TGF-β1 in GO orbital fibroblasts. Preincubation with 200 μM NAC significantly inhibited 6.25 μM H_2_O_2_-induced elevations of intracellular IL-1β and TGF-β1 in the GO orbital fibroblasts (p<0.05 and p<0.001, respectively). A significant reduction in H_2_O_2_-induced elevations of intracellular IL-1β and TGF-β1 was also obtained after the cells were preincubated with 500 μM vitamin C (p<0.005 and p<0.001, respectively).

### Low concentration of hydrogen peroxide–induced changes of reactive oxygen species in Graves’ ophthalmopathy orbital fibroblasts

**Figure 5:**
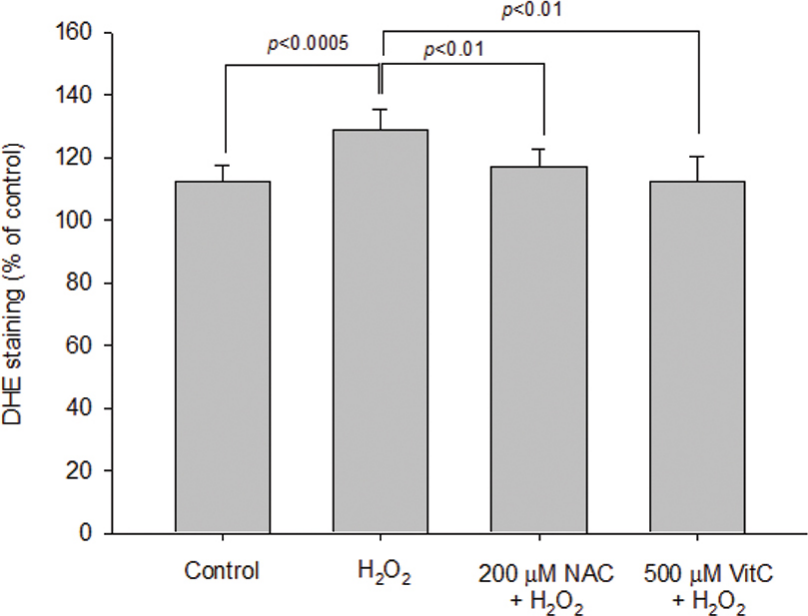
Protective effect of N-acetylcysteine and vitamin C against hydrogen peroxide–induced elevation of superoxide anion production in Graves’ ophthalmopathy orbital fibroblasts. After pretreatment of Graves’ ophthalmopathy (GO) orbital fibroblasts (n=7) with 200 μM N-acetylcysteine (NAC) or 500 μM vitamin C (VitC) for 1 h, followed by the addition of 6.25 μM hydrogen peroxide (H_2_O_2_) for 24 h, the intracellular levels of the superoxide anions were determined with dihydroethidine staining with flow cytometry. The mean values of the superoxide anions from the GO orbital fibroblasts are shown in the histogram. The H_2_O_2_-induced intracellular levels of the superoxide anions in the GO orbital fibroblasts were significantly abolished by pretreatment with 200 μM NAC or 500 μM VitC. The data are presented as mean ± standard deviation of the results from three independent experiments. (p<0.0005, and p<0. 01 versus the indicated group.)

Table 2 shows the 6.25 μM H_2_O_2_ treatment led to significant elevation in the levels of superoxide anions (mean increase: 14.7%, p=0.00015), but not the intracellular H_2_O_2_ content in GO orbital fibroblasts (p=0.076). In addition, when we pretreated the GO orbital fibroblasts with 200 μM NAC or 500 μM vitamin C, the low dose of H_2_O_2_ (6.25 μM)-induced production of the superoxide anions was abolished (Figure 5).

**Table 2:** Intracellular levels of reactive oxygen species in orbital fibroblasts before and after treatment of the cells with 6.25 μM H_2_O_2_

Reactive oxygen species	Before treatment (mean ± SD)	After treatment (mean ± SD)	Induction ratio (%)* (mean ± SD)	*p* -value
H_2_O_2_ (Relative ratio**)				
Normal	101.15±3.46	103.64±6.32	102.49±2.45	0.195
GO	118.56±4.60	117.70±5.52	98.80±3.60	0.587
	p<0.001		p=0.076	
O_2_^.-^ (Relative ratio**)				
Normal	103.26±5.02	104.60±5.19	101.31±1.24	0.477
GO	112.25±5.49	128.80±6.80	114.74±3.41	<0.001
	p<0.001		p<0.001	

* Induction ratio=O_2_^.-^ or H_2_O_2_ value after H_2_O_2_ treatment / baseline value (%) ** Each measurement of cultured orbital fibroblasts was presented as a relative value, which was calculated by taking the intracellular ROS levels of N1 as 100%.

## Discussion

Orbital fibroblasts, one of the major cells affected by GO, contribute to many GO-associated pathologic conditions, including cellular proliferation [15]. For the first time, we demonstrated in this study that GO fibroblasts are hypersensitive not only to high concentrations of H_2_O_2_ but also to low levels of H_2_O_2_. Interestingly, low concentrations of H_2_O_2_ stimulated the proliferation of GO orbital fibroblasts but had little effect on the normal controls. The observation in this study of a biphasic effect of ROS on cellular proliferation is consistent with the findings in various cell types [16,17]. Although how human cells respond biochemically to low concentrations of ROS is not well understood, it has been shown that ROS play a role in signal transduction pathways as a second messenger involving cellular growth and protection of cells against apoptosis [18,19]. In addition, recent data also revealed that the H_2_O_2_ is an important intermediate downstream of adenosine triphosphate receptor pathways leading to enhanced cell proliferation of skeletal myoblasts [20].

Apart from enhanced proliferation of GO orbital fibroblasts, our study also shows that a low level of H_2_O_2_ induced higher intracellular levels of TGF-β1 and IL-1β than those in the normal controls. We also observed increased production of superoxide anion in GO orbital fibroblasts after the 6.25 μM H_2_O_2_ treatment. Moreover, the low dose of H_2_O_2_-induced elevation of the superoxide anions was abolished by the antioxidant treatment. Therefore, we speculate that the increase of TGF-β1 and IL-1β due to the low dose of H_2_O_2_ is related to the formation of superoxide anions in GO orbital fibroblasts. This result is in line with previous observations in other cell types that had demonstrated that oxidative stress is an important modulator of TGF-β and IL-1β expression [21-23]. TGF-β1, a potent fibrogenic cytokine, has been reported to modulate proliferation of fibroblasts and tissue fibrosis [24,25]. IL-1β is known to stimulate hyaluronan synthesis in orbital fibroblasts [26,27]. Hyaluronan accumulation and fibroblast proliferation are important pathological features in the overt expression of ophthalmopathy in patients with GO. Collectively, these findings suggest that ROS may contribute to the pathogenesis of GO either by acting directly or inducing the release of proinflammatory cytokines.

We previously revealed that GO orbital fibroblasts have accumulated higher basal content of ROS such as superoxide anions and H_2_O_2_ compared with those of normal controls [10]. Burch et al. also demonstrated that superoxide anions induce the cellular proliferation of cultured GO orbital fibroblasts [12]. In combination with our current observations of biphasic effects of H_2_O_2_ on the cellular proliferation of GO orbital fibroblasts, we suggest that low levels of ROS may stimulate cellular proliferation and induce more proinflammatory cytokines on GO fibroblasts which promote the development of early GO. Furthermore, accumulating ROS can elicit more oxidative damage and redox imbalance in GO orbital fibroblasts, which further exacerbate existing GO [15]. Therefore, early blockage of ROS formation in orbital fibroblasts may be important in treating or preventing GO. In a small trial, oral antioxidants showed encouraging results in treating mild and moderately severe GO [28]. Recently, selenium (an antioxidant) was successfully applied in patients with mild GO in a large, multicenter, randomized, placebo-controlled trial in Europe [29]. Antioxidants may exert their actions through antioxidative or anti-inflammatory effects. Selenium is an important constituent of the enzyme glutathione peroxidase and thioredoxin reductase, which are responsible for destroying H_2_O_2_ and lipid-damaging peroxides that are increasingly produced in GO [30]. In addition, selenium also may reduce H_2_O_2_-mediated expression of cyclooxygenase-2, which has been reported to be related to the disease activity in Graves' ophthalmopathy [31,32]. Moreover, selenium could decrease the formation of proinflammatory cytokines, especially the T helper type 1 cytokines, which are predominant early in GO [33-35]. In this study, pretreatment with antioxidants effectively ameliorated the effects of low levels of H_2_O_2_ on cellular proliferation and induction of proinflammatory cytokines in GO orbital fibroblasts, further suggesting that antioxidants might have a role in treating early GO and preventing the development of GO.

In conclusion, the biphasic effects of H_2_O_2_ on cellular proliferation of GO orbital fibroblasts may play a role in the pathogenesis of GO. ROS could contribute to the development of GO either by acting directly or inducing the release of proinflammatory cytokines. Most importantly, we demonstrated that pretreatment with antioxidants can ameliorate the cellular proliferation and the induction of proinflammatory cytokines release on GO orbital fibroblasts in response to low levels of oxidative stress. These findings have provided a theoretical basis for the rational use of antioxidants in treating GO at an early stage.
